# Overlapping type 2, barrier, and remodeling endotypes shape clinical expression in eosinophilic esophagitis

**DOI:** 10.3389/fimmu.2026.1948419

**Published:** 2026-09-07

**Authors:** Adam Wawrzeńczyk, Katarzyna Napiórkowska-Baran, Marta Tykwińska, Maciej Szota, Zbigniew Bartuzi, Józef Sławatycki, Alina Kanikowska, Krzysztof Pałgan

**Affiliations:** 1 Department of Allergology, Clinical Immunology and Internal Diseases, Collegium Medicum in Bydgoszcz, Nicolaus Copernicus University in Toruń, Bydgoszcz, Poland; 2 Student Research Club of Clinical Immunology, Department of Allergology, Clinical Immunology and Internal Diseases, Collegium Medicum in Bydgoszcz, Nicolaus Copernicus University in Toruń, Bydgoszcz, Poland; 3 Department of Gastroenterology, Dietetics and Internal Diseases, Poznan University of Medical Sciences, Poznan, Poland

**Keywords:** clinical phenotypes, eosinophilic esophagitis, epithelial barrier, fibrostenosis, IL-13, molecular endotypes, precision medicine, type 2 immunity

## Abstract

Eosinophilic esophagitis (EoE) is a chronic, immune-mediated disease of the esophagus in which symptoms, histologic activity, endoscopic severity, epithelial dysfunction, and fibrostenotic remodeling frequently diverge. Peak eosinophil density remains central to diagnosis and response assessment, but it represents only one component of a broader epithelial–immune and structural process. This review integrates epithelial alarmins, local type 2 immunity, IL-13-driven epithelial programming, eosinophil–mast-cell effector activity, and stromal remodeling within an endotype-to-phenotype framework. We propose that inflammatory, barrier-dominant, relapsing, treatment-responsive, fibrostenotic, and mixed clinical patterns reflect overlapping programs rather than discrete diseases. The strength of evidence differs across the framework: IL-13-associated epithelial activity, eosinophil–mast-cell injury, and remodeling pathways are supported by human tissue, treatment, and functional studies, whereas alarmin-dominant, barrier-relapse, and biomarker-matched treatment constructs remain largely mechanistic or hypothesis-generating. Eosinophils remain biologically active effector cells, but their number alone does not capture degranulation, mast-cell activity, epithelial repair, or accumulated structural injury. We therefore distinguish symptomatic, histologic, endoscopic, molecular, and structural or functional remission and emphasize the complementary roles of EREFS, broader histologic scoring, and structural assessment. The proposed model is a conceptual synthesis of published evidence, not a validated routine treatment-selection algorithm, but it may support future longitudinal validation and stratified research.

## 1 Introduction

Eosinophilic esophagitis (EoE) is a chronic, immune-mediated esophageal disorder characterized by symptoms of esophageal dysfunction and eosinophil-predominant inflammation of the squamous epithelium. Current diagnostic frameworks define EoE by compatible symptoms and at least 15 eosinophils per high-power field on esophageal biopsy, after exclusion of other causes of esophageal eosinophilia (1, 2). Although this definition provides a practical diagnostic standard, it simplifies a biologically heterogeneous disease. Clinically, EoE may present with dysphagia, food impaction, chest discomfort, adaptive eating behaviors, feeding difficulties, or failure to thrive, while endoscopic and histologic findings range from subtle inflammation to rings, strictures, and narrow-caliber esophagus. This diversity shows that EoE cannot be understood solely as an eosinophil-count disorder.

In this review, phenotype denotes the observable clinical expression of disease, including symptoms, endoscopic appearance, structural impairment, disease course, and treatment response. Endotype denotes a biologically coherent mechanism or pathway that contributes to that expression. These terms are not interchangeable: one phenotype may arise from several overlapping endotypes, and one endotype may produce different phenotypes according to age, disease duration, treatment exposure, and remodeling burden.

The imperfect relationship among symptoms, histology, endoscopy, and remodeling remains a central challenge. Patients may remain symptomatic despite histologic improvement, whereas substantial eosinophilic inflammation may coexist with modest symptoms, particularly in children and in patients who adopt compensatory eating behaviors. Rings, strictures, and impaired distensibility reflect cumulative injury and may persist after mucosal inflammation improves. Longitudinal pediatric data and persistent mast-cell activity during eosinophil-defined remission further indicate that these domains evolve on partly independent timelines (3, 4).

At the molecular level, EoE is dominated by type 2 immune activation. Early transcriptomic and cytokine studies identified a disease-specific esophageal inflammatory program marked by local overexpression of IL-13, IL-5, and downstream chemokines such as eotaxin-3/CCL26 (5, 6). IL-13 has emerged as a central epithelial-programming cytokine linking immune activation to barrier dysfunction, chemokine induction, and remodeling pathways. IL-5 supports eosinophil survival and activation, while IL-4 and IL-13 converge through shared receptor signaling to amplify type 2 inflammation. However, type 2 immunity is not uniform. Multisite molecular studies show that patients with similar eosinophil counts may differ in cytokine expression, epithelial gene dysregulation, endoscopic severity, and treatment response (7, 8). Single-cell studies further support this compartmentalized view by identifying clonally expanded, esophagus-homing pathogenic effector Th2 populations, indicating local antigen-driven T-cell responses beyond systemic atopy alone (9).

The distinction between eosinophilic inflammation and broader immune-pathologic activity is clinically important. Molecular endotyping studies have identified EoE subgroups with different combinations of inflammatory activity, epithelial dysfunction, and fibrostenotic features despite comparable eosinophil density (7). These findings suggest that peak eosinophil counts may represent a shared endpoint of biologically distinct immune programs rather than a sufficient disease descriptor. Studies also show persistent molecular abnormalities in histologic remission. Inactive EoE may retain epithelial dysregulation, including periostin accumulation and desmoglein-1 downregulation, even when eosinophil counts fall below the diagnostic threshold (10). Thus, histologic remission does not necessarily equal molecular or structural remission.

Clinical heterogeneity is also shaped by interactions between local esophageal immunity and broader atopic background. Many patients with EoE have concomitant allergic disease, but sensitization patterns do not directly define disease-driving triggers or predictable phenotypes. Component-resolved sensitization studies show substantial variability in allergen recognition, supporting the view that systemic allergic profiles are only one layer of a complex immune landscape (11). This is relevant when classifying patients by dietary response, inflammatory activity, or fibrostenotic risk. Rather than separating EoE into purely “allergic” and “non-allergic” forms, current evidence supports a model in which epithelial barrier dysfunction, antigen-specific type 2 immunity, effector-cell activation, and remodeling pathways combine in varying proportions.

The evolving concept of EoE parallels other chronic inflammatory diseases, where clinical phenotypes are increasingly interpreted through underlying endotypes. In EoE, visible phenotypes such as inflammatory, fibrostenotic, treatment-responsive, or mixed disease may reflect dominant but overlapping immunologic programs. An IL-13-high epithelial endotype may drive inflammatory and barrier-disruptive features; an eosinophil- and mast-cell-dominant effector pattern may contribute to symptoms and mucosal injury; and a remodeling-dominant program involving periostin, TGF-β-related pathways, endothelial–fibroblast crosstalk, and extracellular matrix deposition may promote fibrostenotic transformation (12–14). These programs are not mutually exclusive and may evolve over time, especially when chronic inflammation remains untreated or incompletely controlled.

In this review, we synthesize evidence linking type 2 immune pathways to clinical phenotypes in EoE. We focus on how epithelial-derived alarmins, Th2 cytokines, eosinophils, mast cells, barrier proteins, and remodeling mediators shape disease expression. We then propose an endotype-to-phenotype framework to explain the frequent dissociation between symptoms, histology, molecular inflammation, and structural remodeling. We argue that the clinical heterogeneity of EoE is best understood as the surface expression of overlapping and evolving immunologic and structural endotypes, with IL-13-driven epithelial programming providing a central mechanistic bridge between type 2 immunity and disease expression.

### 1.1 Conceptual advances of this review

Previous studies have characterized type 2 cytokine signaling, epithelial barrier dysfunction, eosinophilic and mast-cell inflammation, and tissue remodeling. The present review connects these components within one dynamic framework while retaining a clear distinction between observable phenotype and proposed biological endotype.

The model integrates IL-13-driven epithelial programming, alarmin-mediated activation, eosinophil–mast-cell effector circuits, and stromal–vascular remodeling. These modules overlap and may change over time; the model therefore does not assign each patient to a fixed subtype.

The best-established evidence comes from molecular clustering, human tissue studies, treatment trials, endoscopic and functional cohorts, and longitudinal remodeling data. By contrast, the proposed alarmin-dominant, barrier-relapse, and treatment-matched modules should be interpreted as mechanistic or hypothesis-generating constructs requiring prospective validation.

A similar distinction between visible morphology and underlying mechanism is relevant across gastrointestinal mucosal disorders, in which comparable structural or histologic abnormalities can arise from immune, infectious, genetic, or iatrogenic pathways (15).

Accordingly, the framework is intended to organize evidence, identify testable hypotheses, and support phenotype-aware interpretation; it is not presented as a validated molecular algorithm for routine treatment selection.

### 1.2 Literature selection approach

This article is a narrative, mechanism-oriented review rather than a systematic review or meta-analysis. Literature was identified through targeted searches of PubMed/MEDLINE and Google Scholar, supplemented by reference screening of relevant guidelines, consensus documents, key clinical studies, translational papers, molecular endotyping studies, and major reviews. Priority was given to human tissue studies, multicenter cohorts, controlled trials, tissue-based transcriptomic and single-cell studies, molecular endotyping research, and recent publications. Seminal mechanistic studies were included when essential to the conceptual framework. No formal PRISMA-based selection, risk-of-bias assessment, or quantitative synthesis was performed. Experimental findings were treated as hypothesis-generating unless supported by human tissue or clinical data. The proposed framework is a conceptual synthesis of published literature and contains no unpublished clinical, experimental, or molecular data.

## 2 Type 2 immunity as the core driver of EoE

Eosinophilic esophagitis is traditionally identified by epithelial eosinophilia, but its immunologic architecture extends beyond eosinophil accumulation alone. Current evidence supports a model in which the esophageal epithelium acts not only as a target of inflammation, but also as an active immune interface that senses environmental stimuli, releases alarmins, shapes local type 2 immunity, and drives downstream effector and remodeling pathways. In this framework, eosinophils remain a visible and important component of EoE, but they function within a tissue-specific epithelial–immune circuit involving alarmins, Th2 cells, ILC2s, and IL-13-dominant epithelial programming.

Disease initiation is closely linked to epithelial barrier disruption. The esophageal mucosa is exposed to food antigens, aeroallergen-derived proteins, acid, mechanical stress, and microbial or viral stimuli. In susceptible individuals, impaired barrier integrity permits antigen penetration and activation of epithelial stress pathways. Evidence of dust mite antigen penetration into the esophageal epithelium in active EoE supports local aeroallergen-related immune activation rather than systemic sensitization alone (16). This is consistent with the broader concept that EoE-associated immune activity is compartmentalized within esophageal tissue.

Epithelial-derived alarmins are central to local immune initiation. IL-33 is increased in EoE mucosa and functionally linked to EoE-like inflammation in experimental systems (17, 18). In epithelial IL-33 overexpression models, IL-33 induces basal-zone hyperplasia, eosinophilic infiltration, mast cell accumulation, and tissue remodeling through ST2-dependent pathways. Genetic deletion of IL-13 abrogates this phenotype, placing IL-33 upstream of the IL-13 axis (17). These findings shift the conceptual starting point of EoE from eosinophil recruitment to epithelial danger signaling.

TSLP provides another epithelial route into type 2 immunity. Esophageal epithelial cells preferentially secrete TSLP when differentiated, especially in suprabasal layers, and TSLP production can be induced by innate immune stimuli such as TLR3 activation and selected food antigens (19). Thus, epithelial differentiation state may determine the ability to generate type 2–polarizing signals. In EoE, epithelial dysfunction may therefore weaken the barrier while also altering tissue immune tone through dendritic cell activation, Th2 polarization, and ILC2 expansion. IL-25 is less consistently characterized than IL-33 and TSLP, but belongs to the same alarmin network linking epithelial stress to type 2 amplification.

Downstream of alarmins, type 2 cytokines define the dominant inflammatory program. Local overexpression of IL-13, IL-5, IL-4, and eotaxin-3/CCL26 has been repeatedly shown in EoE tissue (5, 6). IL-13 is central because it connects immune activation with epithelial remodeling. It induces CCL26, suppresses epithelial differentiation genes, alters barrier proteins, and drives a transcriptomic program overlapping with the EoE disease signature (5, 20, 21). IL-5 mainly supports eosinophil recruitment, survival, and activation, but local expression appears more relevant than systemic cytokine levels. Experimental data showing esophageal remodeling in tissue-specific IL-5-driven eosinophilia support the role of IL-5 in eosinophil-mediated injury while emphasizing the importance of local cytokine context (22).

Genetic and molecular evidence further supports the tissue specificity of EoE. Genome-wide association studies identified susceptibility loci containing genes enriched for esophageal expression, particularly CAPN14, an esophagus-specific gene regulated by IL-13 (23). This is difficult to reconcile with EoE as a generic systemic eosinophilic disorder. Instead, it suggests that disease risk is partly determined by esophageal epithelial biology. Esophagus-specific CAPN14 expression and IL-13 responsiveness help explain why type 2 inflammation selectively manifests in the esophageal mucosa rather than uniformly across the gastrointestinal tract.

Innate lymphoid cells add another layer to this epithelial–immune model. Esophageal ILC2s can contribute directly to epithelial remodeling through amphiregulin–EGFR signaling (24). In experimental systems, ILC2-derived amphiregulin promotes basal cell proliferation and epithelial thickening, while blockade of amphiregulin or EGFR signaling attenuates EoE-like pathology. Epithelial defects may persist in eosinophil-deficient settings but disappear when ILC-dependent pathways are disrupted, suggesting that ILC2s may lie upstream in epithelial remodeling. These findings link epithelial alarmin release to structural changes previously attributed mainly to chronic eosinophilic inflammation.

Adaptive type 2 immunity also shows local antigen-driven organization. Single-cell RNA and T-cell receptor sequencing identified clonally expanded pathogenic effector Th2 cells in EoE, including GPR15-expressing populations enriched in esophageal tissue and among milk-reactive T cells (9). This supports a model in which antigen-specific Th2 cells home to, expand within, and maintain the esophageal inflammatory niche. Such tissue-associated Th2 populations argue against a purely systemic allergic process and help explain why peripheral biomarkers often fail to reflect disease activity.

Eosinophils are indispensable diagnostic and biologic components of EoE, but they are not necessarily the initiating or rate-limiting mechanism. IL-13 can induce epithelial reprogramming and remodeling through eosinophil-independent pathways, whereas anti-IL-5 therapy can markedly reduce tissue eosinophilia without consistently normalizing symptoms or structural disease. Eosinophils should therefore be positioned as active downstream effectors recruited by epithelial–type 2 circuits: their degranulation, cytokine release, and interactions with mast cells and fibroblasts amplify injury, but eosinophil density alone does not explain disease initiation, persistence, or fibrostenotic progression (25).

Type 2 heterogeneity further limits eosinophil-centered interpretation. Multisite molecular profiling has shown that patients with active EoE cluster into distinct type 2 cytokine-expression groups differing in age, molecular score, and remodeling-associated signatures, without proportional differences in eosinophil density (8). Thus, similar eosinophil counts may reflect different upstream immune programs. Some patients may have a dominant IL-13 epithelial signature, others stronger IL-5/eosinophil activation, and others remodeling-related programs only partly linked to current inflammation. This molecular heterogeneity helps explain why symptoms, histology, and structural abnormalities often diverge.

Collectively, the evidence supports a tissue-specific sequence in which epithelial susceptibility and barrier stress promote alarmin-driven Th2 and ILC2 activity, IL-13 and IL-5 signaling, eosinophil recruitment, epithelial dysfunction, and remodeling. Different configurations of this network may generate inflammatory, treatment-responsive, mixed, or fibrostenotic presentations.

Type 2 immunity remains the dominant organizing axis, but clinical expression also reflects genetic susceptibility, intrinsic barrier defects, epithelial repair, stromal–vascular remodeling, smooth-muscle dysfunction, and altered distensibility. Once these structural programs are established, fibrostenotic disease may become partly uncoupled from current mucosal eosinophilia (10, 12–14, 23).

## 3 IL-13-driven epithelial programming: the molecular bridge between type 2 immunity and disease expression

Among type 2 cytokines implicated in eosinophilic esophagitis, IL-13 occupies a central position because it converts immune activation into epithelial dysfunction, chemokine production, and early remodeling. Rather than acting only as an inflammatory amplifier, IL-13 reprograms the esophageal epithelium into an active disease compartment. This links upstream type 2 immunity to eosinophil recruitment, impaired barrier integrity, basal-zone hyperplasia, altered differentiation, and fibrostenotic risk.

Early transcriptomic studies established IL-13 as a dominant driver of the EoE molecular signature. IL-13 expression is increased in active EoE tissue, and exposure of primary esophageal epithelial cells to IL-13 induces a gene-expression profile that substantially overlaps with the disease transcriptome in patient biopsies (26). This signature includes inflammatory and epithelial-structural genes, showing that IL-13 does not merely recruit eosinophils but reshapes epithelial identity. Glucocorticoid treatment can reverse much of this program, suggesting that epithelial reprogramming is dynamic and treatment-responsive (26).

A major downstream effect of IL-13 is epithelial chemokine induction. IL-13 potently induces eotaxin-3/CCL26 through STAT6-dependent mechanisms, directly linking type 2 cytokine signaling to eosinophil recruitment (5, 26). CCL26 is among the most strongly upregulated epithelial transcripts in EoE and correlates with local eosinophilic inflammation. This explains why eosinophils are a reproducible histologic feature, while placing eosinophilia downstream of the IL-13–epithelial axis rather than at the origin of disease.

IL-13 also suppresses terminal epithelial differentiation. Studies of the epithelial differentiation complex show coordinated downregulation of filaggrin, involucrin, and related barrier genes in EoE, with IL-13 reproducing many of these changes in epithelial models (21). Because the esophageal epithelium depends on regulated squamous differentiation for barrier function, loss of differentiation proteins increases permeability, facilitates antigen penetration, and sustains immune activation. Experimental models also show that IL-13 alters keratin programs, including downregulation of differentiation-associated keratins such as KRT78, linking type 2 inflammation to a less mature and more injury-prone epithelial state (27).

Barrier dysfunction results from converging abnormalities in desmosomal adhesion, tight junction organization, cytoskeletal regulation, and epithelial differentiation. Filaggrin deficiency functionally impairs epithelial resistance, while IL-13 alters tight junction localization and organization even when total protein abundance is not uniformly reduced (28). Thus, epithelial barrier failure in EoE may reflect junctional mislocalization and disorganization rather than loss of a single tight junction protein, meaning standard histology may underestimate functional impairment.

Desmoglein-1 is one of the clearest examples of IL-13-driven epithelial reprogramming causing structural dysfunction. DSG1 is reduced in EoE biopsies, and experimental DSG1 silencing weakens epithelial integrity, induces intercellular separation, and produces transcriptional changes overlapping with the EoE molecular profile (29). DSG1 loss also promotes periostin expression, linking barrier disruption to extracellular matrix remodeling and helping explain why epithelial dysfunction and remodeling may persist even after eosinophil counts fall.

CAPN14 provides a tissue-specific link between IL-13 and epithelial fragility. Identified as an EoE-associated, esophagus-enriched gene, CAPN14 is strongly induced by IL-13 and directly contributes to impaired epithelial barrier function (23, 30). CAPN14 can disrupt epithelial architecture and promote loss of DSG1-dependent cohesion, connecting genetic susceptibility, IL-13 responsiveness, and barrier breakdown in one pathway (30). Its relative esophageal specificity also helps explain why a systemic tendency toward type 2 immunity manifests as localized esophageal disease.

Beyond barrier impairment, IL-13 contributes to basal-zone hyperplasia. ANO1, a calcium-activated chloride channel, is increased in the basal epithelium and functions as a driver of epithelial proliferation and basal-zone expansion (31). IL-13 also activates STAT3-dependent signaling, with SFRP1 acting as a downstream regulator of epithelial proliferation and inflammatory remodeling (32). These data broaden the IL-13 model beyond STAT6 alone: STAT6 remains central for canonical type 2 gene expression and chemokine induction, while STAT3- and proliferation-associated pathways contribute to architectural disease changes.

Cytoskeletal remodeling adds another layer of epithelial dysfunction. Synaptopodin, an actin-associated protein, is induced by IL-13 and regulates epithelial motility, differentiation, and barrier integrity (33). Thus, IL-13 modifies not only immune signaling and junctional proteins but also epithelial cell mechanics, potentially affecting repair, wound healing, and restitution after inflammatory injury.

Therapeutic data reinforce the clinical relevance of IL-13-driven epithelial programming. Anti-IL-13 treatment can reverse epithelial–mesenchymal transition biomarkers and improve molecular remodeling features in active EoE (34). Cendakimab data further show that IL-13 blockade improves esophageal gene-expression abnormalities related to IL-13 signaling, epithelial differentiation, mast cell activity, and remodeling (35). These findings support a causal role for IL-13 in maintaining the epithelial disease program. Molecular improvement despite incomplete clinical or endoscopic responses suggests that symptoms and structural outcomes may also depend on downstream or partly irreversible components, especially in established fibrostenotic disease (36).

The epithelial response to IL-13 also helps explain phenotype heterogeneity. In inflammatory disease, IL-13-driven CCL26 production and barrier dysfunction may dominate, producing eosinophilic inflammation, furrows, exudates, and treatment-responsive activity. In barrier-dysfunction phenotypes, persistent DSG1, filaggrin, CAPN14, or tight junction abnormalities may sustain antigen penetration and relapse risk despite partial histologic improvement. In remodeling-prone disease, IL-13-induced periostin, epithelial–mesenchymal transition, and endothelial–fibroblast crosstalk may contribute to rings, strictures, and reduced distensibility (12, 14, 34). These phenotypes are not mutually exclusive, but reflect different weights of the same epithelial–immune program.

IL-13-driven epithelial programming links type 2 immunity to several partially independent outputs: CCL26-mediated eosinophil recruitment, CAPN14- and DSG1-associated loss of cohesion, impaired differentiation, basal-zone hyperplasia, and periostin/EMT-related remodeling. These outputs can improve at different rates, explaining why peak eosinophil density does not fully represent epithelial or structural recovery.

**Figure 1:**
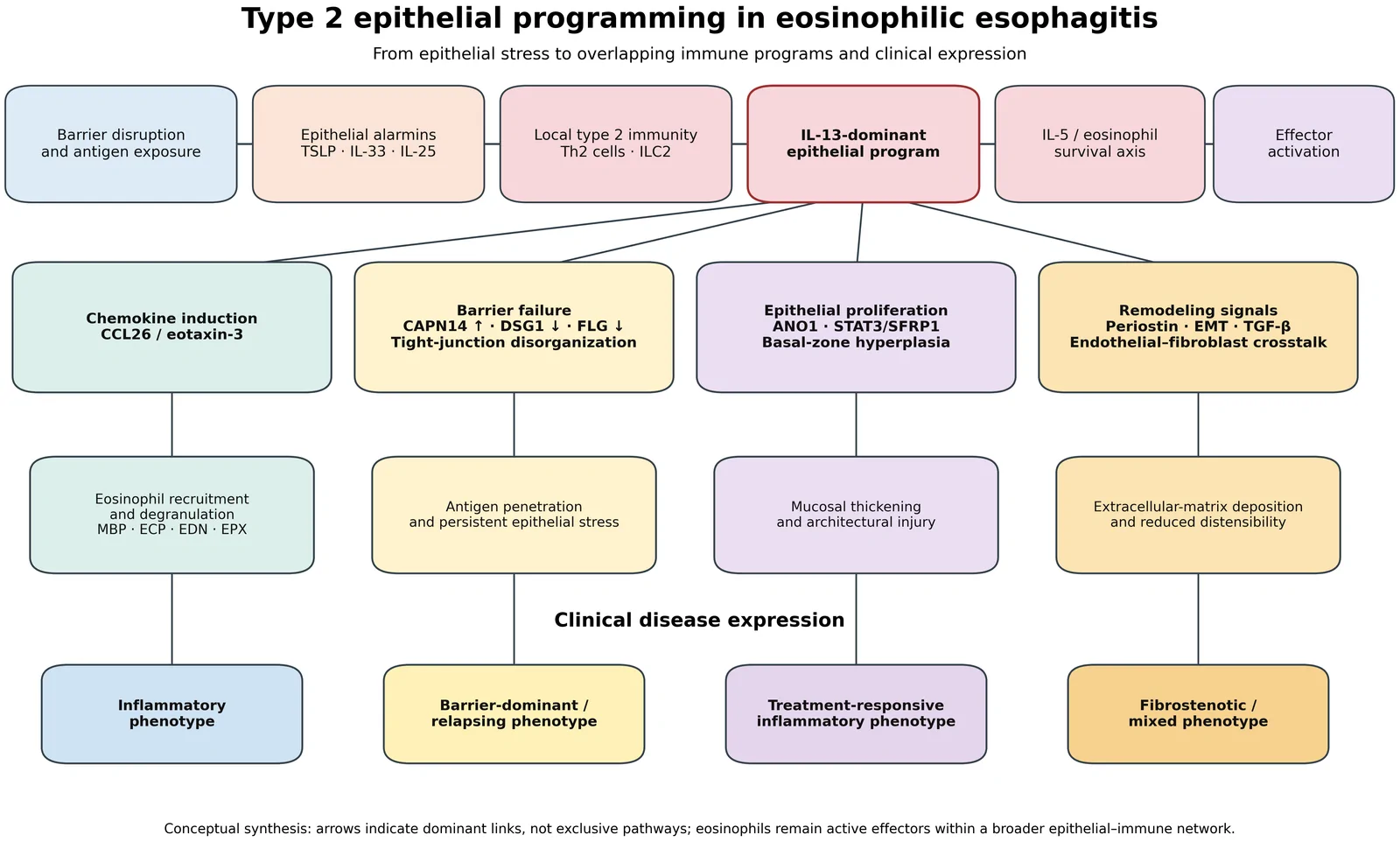
Type 2 epithelial programming in eosinophilic esophagitis. Barrier stress activates epithelial alarmins and local Th2/ILC2 pathways. IL-13-centered epithelial programming promotes CCL26-mediated eosinophil recruitment, barrier failure, basal-zone hyperplasia, and remodeling. The pathways overlap, and eosinophils remain active downstream effectors rather than passive markers.

The epithelial consequences of type 2 immune activation are summarized in **Figure 1**, which conceptualizes IL-13-driven epithelial programming as the mechanistic bridge linking epithelial alarmin release and local Th2/ILC2 responses with eosinophil recruitment, barrier failure, basal-zone hyperplasia, and remodeling-associated clinical phenotypes.

## 4 Effector inflammation: eosinophils, mast cells, and tissue injury

The epithelial IL-13 program creates a permissive inflammatory environment, but tissue injury is produced by several effector compartments. Eosinophils remain the defining histologic feature and contribute to tissue injury by releasing cytotoxic granule proteins and fibrogenic mediators and by promoting neuromuscular dysfunction. Mast cells, subepithelial inflammation, extracellular granule deposition, and persistent epithelial abnormalities can nevertheless sustain symptoms and tissue injury when intact eosinophil counts fall below conventional thresholds.

Eosinophils are recruited mainly downstream of epithelial type 2 signaling. IL-13-induced CCL26/eotaxin-3 promotes chemotaxis, while IL-5 supports eosinophil survival, activation, and tissue persistence. Once recruited, eosinophils release cytotoxic granule proteins, including major basic protein, eosinophil cationic protein, eosinophil-derived neurotoxin, and eosinophil peroxidase. These mediators injure epithelial cells, increase permeability, activate fibroblasts, and amplify inflammation. Thus, eosinophils are not passive markers, but active effector cells translating type 2 immunity into tissue damage.

Eosinophil-derived products also contribute to remodeling. In pediatric EoE, epithelial–mesenchymal transition has been linked to eosinophilic inflammation and subepithelial fibrosis, with treatment-related improvement paralleling reduced inflammatory activity (37). Experimental and translational studies show that Th2 cytokines, TGF-β1, and eosinophil products can induce fibrogenesis in esophageal fibroblasts and alter muscle function (38). These findings support a model in which eosinophils participate in the shift from mucosal inflammation to deeper tissue dysfunction, especially when inflammation persists.

Standard histology enumerates morphologically intact intraepithelial eosinophils but incompletely captures degranulation, extracellular granule-protein deposition, and deeper-wall inflammation. Major basic protein deposition can correlate with symptoms independently of peak eosinophil counts and may persist below the diagnostic threshold (39). The peak eosinophil count, expressed as eosinophils per high-power field (eos/hpf), is therefore an essential measure of inflammatory burden, but not a complete measure of eosinophil activity or tissue injury.

Clinical trials targeting IL-5 confirm the importance of eosinophils while also defining the limits of eosinophil depletion as a therapeutic endpoint. Reduced eosinophilia has produced incomplete and variable symptom improvement, consistent with residual barrier dysfunction, mast-cell activity, neurosensory changes, or established remodeling (25, 40).

Mast cells are among the best-characterized non-eosinophil effector populations in EoE. Their infiltration and disease-associated transcriptomic program respond to swallowed corticosteroids and dietary therapy, supporting a role in active mucosal disease rather than incidental bystander activity (41, 42).

The eosinophil–mast cell relationship is bidirectional. In a pediatric anti-IL-5 study, mepolizumab reduced eosinophils, mast cells, IL-9-positive cells, and mast cell–eosinophil couplets (43). Because eosinophils were identified as a source of IL-9, they may support esophageal mastocytosis. Conversely, mast cells can produce type 2 and profibrotic mediators, including IL-13 and TGF-β-related signals, sustaining inflammation and remodeling. Eosinophils and mast cells therefore form a partially interdependent effector circuit.

Single-cell data further refine this model. Mast cells in EoE are heterogeneous, locally proliferative, and dynamically activated across disease states (44). Active EoE contains pro-inflammatory mast cell populations, while activated mast cell subsets may persist during histologic remission and remain poised to reinitiate inflammation. This is relevant to the symptom–histology disconnect, because residual mast cell activation may maintain epithelial dysfunction, mediator release, and sensory activation despite normalized eosinophil counts.

Mast cells may also directly contribute to symptoms. In pediatric cohorts, mast cell density has been associated with persistent symptoms and endoscopic abnormalities despite resolution of eosinophilia (4). In adults, symptomatic EoE may differ from asymptomatic esophageal eosinophilia by higher mast cell tryptase staining and increased expression of mast cell-related receptors such as PAR-2 and VPAC-1, despite similar peak eosinophil counts (45). These findings suggest that mast cell mediators influence symptom perception through epithelial, neuroimmune, and smooth muscle pathways.

The smooth muscle compartment provides another link between mast cells and clinical expression. Mast cells infiltrate esophageal smooth muscle in EoE, express TGF-β1, and can increase smooth muscle contraction *in vitro* (46). Dysphagia and food impaction therefore reflect not only epithelial inflammation, but also altered tissue stiffness, motility, and remodeling. Mast cell activity in deeper compartments may contribute to symptoms not captured by superficial epithelial eosinophil counts.

Persistent molecular abnormalities also show that tissue injury can outlast eosinophil-defined activity. Histologic remission does not necessarily normalize the esophageal molecular landscape. Persistent periostin accumulation and desmoglein-1 downregulation have been observed in inactive disease and even deep remission, indicating incomplete restoration of epithelial and structural integrity (10). These findings suggest that once barrier dysfunction, extracellular matrix remodeling, or mast cell activation is established, eosinophil reduction may be necessary but insufficient for full disease resolution.

Eosinophils and mast cells are complementary effector modules within the epithelial–type 2 network. Eosinophils contribute to cytotoxic injury and fibrogenic signaling through degranulation, whereas mast cells contribute mediator release, IL-13-related activity, smooth-muscle dysfunction, and symptom perception. Assessment beyond peak eos/hpf is most relevant when symptoms, endoscopic abnormalities, or structural disease persist despite apparent histologic improvement.

## 5 From inflammation to fibrostenosis: immune mechanisms of remodeling in EoE

Tissue remodeling is a defining feature of EoE and is better understood as an injury–repair continuum than as a simple late consequence of eosinophilia. Barrier damage and type 2 inflammation initiate repair responses involving epithelial cells, eosinophils, mast cells, fibroblasts, endothelial cells, smooth muscle, and extracellular matrix. Persistent signaling can redirect repair toward rings, strictures, luminal narrowing, and reduced compliance (47).

Subepithelial fibrosis was among the earliest recognized remodeling abnormalities in EoE. Pediatric studies showed increased lamina propria fibrosis, vascular activation, and subepithelial remodeling compared with controls, confirming that EoE extends beyond the epithelial layer (48, 49). This is clinically important because routine biopsies incompletely sample the lamina propria and deeper wall compartments, although these areas are central to stricture formation and altered mechanics. Thus, reduced epithelial eosinophilia may not fully reflect ongoing structural disease.

Multiple type 2 pathways converge on fibroblast activation and extracellular matrix deposition. IL-13 and IL-4 stimulate epithelial and stromal cells through JAK–STAT6 signaling, while IL-5 supports eosinophil accumulation and remodeling (22, 50). TGF-β1 provides a parallel profibrotic axis. Translational studies show that Th2 cytokines, TGF-β1, and eosinophil-derived products induce fibronectin and collagen production by esophageal fibroblasts and alter smooth muscle function (38). Remodeling is therefore a multicellular process in which epithelial cytokine signaling recruits inflammation, eosinophil products amplify fibroblast activation, and stromal compartments convert inflammation into stiffness.

Within this continuum, TGF-β-dependent myofibroblast activation and extracellular-matrix accumulation provide a central bridge from persistent tissue injury to fibrostenosis. The process may remain partly reversible during active inflammation but become increasingly self-sustaining as matrix reorganization, vascular-stromal crosstalk, and smooth-muscle dysfunction become established (47).

Epithelial–mesenchymal transition is one mechanism linking mucosal inflammation to fibrosis. In pediatric EoE, EMT markers correlate with eosinophil density, eosinophil peroxidase staining, TGF-β expression, and fibrosis scores, and improve after therapies inducing clinicopathologic remission (37). Anti-IL-13 therapy also reverses EMT biomarkers in adults with active EoE, supporting IL-13 as a driver of epithelial–stromal remodeling (34). These findings suggest that remodeling is not merely a late scar, but can be dynamically regulated by active immune signaling, especially early in disease.

Periostin is another important link between type 2 inflammation and extracellular matrix remodeling. It is induced by IL-13 and TGF-β-related pathways and is strongly upregulated in EoE tissue (14, 51). Functionally, periostin promotes eosinophil adhesion and tissue infiltration, creating a feed-forward loop in which remodeled matrix supports inflammatory cell retention (51). Persistent periostin expression in histologic remission suggests that matrix abnormalities may outlast eosinophil reduction and contribute to barrier dysfunction or relapse risk (10).

Recent studies expand remodeling beyond epithelial and eosinophil compartments. TSPAN12 loss in esophageal endothelial cells is strongly associated with fibrostenotic EoE and promotes endothelial–fibroblast crosstalk, increased permeability, and extracellular matrix dysregulation (12). This identifies a vascular–stromal pathway through which IL-13-driven inflammation may promote fibrostenosis without a direct linear relationship to peak eosinophil counts. In parallel, single-cell and functional studies suggest that EoE fibroblasts may acquire persistent pathogenic programs involving impaired regenerative behavior and abnormal CD73/adenosine-related signaling (52). These data support the idea that advanced remodeling may be maintained by structural cells even after partial inflammatory control.

Clinically, remodeling is best assessed through structural and functional outcomes rather than eosinophil counts alone. Endoscopic rings and strictures reflect chronic remodeling, while reduced distensibility provides a quantitative measure of esophageal rigidity. Functional lumen imaging probe studies show that reduced distensibility correlates with fibrostenotic severity, food impaction risk, and need for dilation (53, 54). Importantly, distensibility does not always correlate with current mucosal eosinophilia, reinforcing the distinction between active inflammation and established structural disease.

Time strongly influences fibrostenotic progression. Diagnostic delay is associated with stricture formation, and fibrotic features increase with longer untreated disease duration (55). Large phenotypic analyses support a natural history in which EoE progresses from inflammatory disease toward mixed or fibrostenotic phenotypes with age and disease duration (56). FLIP-based data further show that reduced distensibility parallels symptom duration and diagnostic delay, providing functional evidence that EoE can behave as a progressive fibrostenotic disorder (57).

Remodeling is not uniformly irreversible. Pediatric studies show that subepithelial fibrosis may improve after topical corticosteroids and dietary elimination, especially when anti-inflammatory treatment begins early (58). Longitudinal data also suggest that histologic response is associated with improved distensibility and symptom burden over time (59). These findings support a two-phase model: in early or active inflammatory disease, remodeling remains partly reversible because it is driven by cytokine signaling, eosinophil activation, and EMT; in advanced fibrostenotic disease, fibrosis, smooth muscle dysfunction, matrix reorganization, and stromal-cell programs may become partly autonomous.

Inflammatory and fibrostenotic phenotypes are therefore biologically connected but not temporally equivalent. Anti-inflammatory therapy can improve early fibrosis and distensibility, whereas established rings, strictures, and mechanical impairment may persist despite histologic response (22, 34, 37, 38, 53–59).

A remodeling-dominant pattern may include an IL-13/periostin epithelial component, eosinophil/TGF-β-associated active fibrogenesis, and a later endothelial–fibroblast, matrix, or smooth-muscle component. Their relative contributions may determine whether disease appears inflammatory, mixed, or predominantly fibrostenotic.

Fibrostenotic transformation thus reflects cumulative immune–structural injury rather than a single contemporaneous eosinophil measurement. Early control of inflammation may limit progression, while delayed diagnosis permits remodeling to become less reversible.

## 6 Clinical phenotypes in EoE: inflammatory, fibrostenotic, treatment-responsive, and mixed disease

Clinical phenotype describes the observable pattern of EoE at a given time, including symptoms, endoscopic findings, histologic activity, structural impairment, disease behavior, and treatment response. The conventional inflammatory, fibrostenotic, and mixed categories remain clinically useful, but they do not constitute a complete biological taxonomy.

### 6.1 Endoscopic assessment and EREFS

Endoscopy remains fundamental for identifying previously unassessed disease and monitoring treatment response. The Eosinophilic Esophagitis Endoscopic Reference Score (EREFS) standardizes five core features—edema, rings, exudates, furrows, and strictures—and separates predominantly inflammatory findings from fibrostenotic abnormalities. The system has demonstrated reproducibility, diagnostic accuracy, and responsiveness to therapy (60, 61).

**Figure 2:**
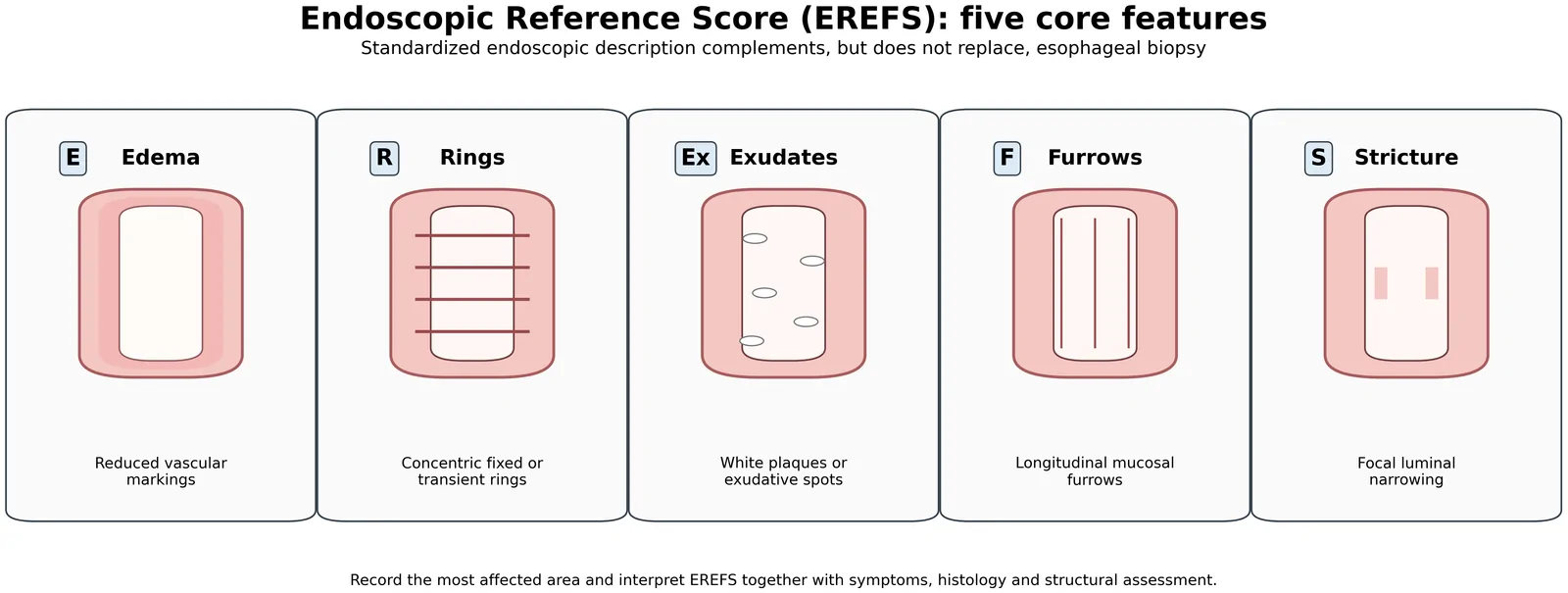
Schematic representation of the eosinophilic esophagitis endoscopic reference score (EREFS). EREFS records edema, rings, exudates, furrows, and strictures in the most affected esophageal area. It standardizes endoscopic description and treatment-response assessment but should be integrated with symptoms, histology, and structural evaluation.

EREFS should be interpreted with biopsy rather than used as a substitute for histology: a macroscopically normal esophagus does not exclude EoE, and endoscopic, symptomatic, and histologic responses may be discordant. Nevertheless, systematic EREFS reporting improves longitudinal comparison and helps identify structural disease that peak eosinophil density cannot capture (62). The five features are summarized schematically in **Figure 2**.

### 6.2 Asymptomatic esophageal eosinophilia

Patients without symptoms of esophageal dysfunction who have compatible endoscopic findings and ≥15 eos/hpf are better described as having asymptomatic esophageal eosinophilia (aEE), because current diagnostic criteria require symptoms for classical EoE. aEE is a related clinicopathologic state rather than a validated EoE endotype. Long-term cohorts show heterogeneous behavior: many patients remain stable or improve, whereas a minority develop symptoms, supporting individualized follow-up rather than automatic classification as symptomatic EoE (63).

The inflammatory phenotype is characterized by edema, furrows, exudates, and active mucosal inflammation. It is more common in children and in patients with shorter disease duration. Pediatric patients often present with nonspecific symptoms such as abdominal pain, vomiting, feeding difficulty, food refusal, or failure to thrive, whereas adolescents increasingly resemble adults with dysphagia and food impaction (64). Inflammatory disease is often more treatment-responsive because remodeling is less established and mucosal inflammation remains more closely linked to active type 2 immunity.

The fibrostenotic phenotype is defined by rings, strictures, luminal narrowing, and reduced esophageal compliance. It predominates in adults and in patients with prolonged untreated disease. Dysphagia and food impaction reflect fixed mechanical obstruction and altered biomechanics rather than mucosal inflammation alone. Phenotypic and natural history studies support a progressive model in which diagnostic delay and persistent inflammation increase the likelihood of fibrostenotic transformation (55, 56). This does not mean all patients inevitably progress, but uncontrolled inflammation over time increases structural risk.

Mixed phenotypes are common and clinically important. Many patients show both inflammatory and fibrostenotic features, such as furrows with rings or strictures. This likely reflects overlapping endotypes: active IL-13/CCL26-driven inflammation may coexist with periostin-, TGF-β-, and fibroblast-mediated remodeling. Mixed disease is therefore not simply an intermediate category, but a complex state in which current inflammation and cumulative structural injury both shape symptoms and treatment response.

Age strongly modifies clinical expression. Children more often show inflammatory features and nonspecific gastrointestinal symptoms, while adults are enriched for fibrostenotic disease and food impaction (64). This gradient likely reflects both biology and time: pediatric EoE may represent earlier, more reversible disease, whereas adult fibrostenosis often reflects longer diagnostic delay and cumulative remodeling. However, age alone is insufficient, as some children develop fibrostenotic features and some adults retain inflammation-predominant disease.

Atopy and sensitization add further complexity. Many patients have allergic rhinitis, asthma, atopic dermatitis, or food sensitization, but allergic status does not map reliably onto endoscopic phenotype or treatment response. Broad sensitization profiles may reflect cross-reactive allergen families rather than true disease-driving triggers, especially in pollen-food or panallergen-related patterns (65). Phenotyping based only on IgE or sensitization therefore risks conflating systemic atopy with local esophageal immune activation.

### 6.3 EoE associated with oral immunotherapy

EoE may emerge or become clinically apparent during oral immunotherapy (OIT), particularly in pediatric practice. Repeated mucosal allergen exposure may promote disease in predisposed patients, although OIT can also unmask previously unrecognized symptoms. A recent systematic review estimated EoE in approximately 1–2% of OIT recipients, with higher pooled estimates for milk and egg than for peanut protocols; substantial heterogeneity and retrospective ascertainment limit causal inference (66). Persistent dysphagia, food refusal, vomiting, or abdominal pain during OIT should prompt evaluation. OIT-associated EoE is best considered a trigger-associated clinical context, not a distinct molecular endotype.

Treatment response is another relevant phenotypic dimension. Pediatric registry data suggest that swallowed topical corticosteroids often achieve higher clinico-histologic response rates than proton pump inhibitors or diet, although outcomes vary by age, phenotype, dose, center practice, and disease characteristics (67). PPI-responsive disease is now best understood as part of the EoE spectrum rather than a separate diagnostic entity (2). It may represent a modifiable phenotype involving epithelial barrier function, acid-related amplification, and type 2 inflammatory pathways.

Eosinophil distribution may further refine treatment-responsive phenotyping. Recent data suggest that isolated distal eosinophilia predicts better histologic response to PPI therapy, whereas proximal-predominant or diffuse eosinophilia may be linked to lower response rates (68). This links a simple histologic pattern to treatment probability without implying that PPI-responsive EoE is biologically separate. Instead, PPI response may identify one configuration of epithelial inflammation within the broader EoE spectrum.

Fibrostenotic disease is generally less responsive to anti-inflammatory therapy alone, especially when narrowing is advanced. Narrow-caliber esophagus and fixed strictures often require dilation in addition to medical therapy. Functional assessment adds another layer: FLIP panometry identifies physiomechanical patterns that do not always correspond to mucosal eosinophilia, and fibrostenotic patterns may predict poor response to pharmacologic therapy alone (13). Thus, endoscopic and functional phenotypes capture chronic remodeling not represented by eosinophil density.

Effective early treatment may modify long-term phenotype. Pediatric follow-up data show that histologic response or endoscopic normalization after initial therapy is associated with lower later fibrostenosis rates (69). This supports the idea that inflammatory and fibrostenotic phenotypes are not fully independent categories, but trajectories shaped by cumulative inflammatory burden. Early inflammation is more reversible, whereas delayed control allows remodeling to become less responsive to immune suppression.

Molecular endotyping further complicates clinical classification. Shoda and colleagues identified EoE endotypes with distinct clinical and histologic patterns despite comparable eosinophil levels: a mild endotype with relatively normal endoscopy, an inflammatory steroid-refractory endotype, and a fibrostenotic adult-onset endotype (7). This shows that inflammatory–fibrostenotic classification is useful but biologically incomplete. A patient may appear inflammatory yet have a steroid-refractory molecular program, or may have fibrostenotic disease driven by remodeling pathways only partly reflected by current mucosal inflammation.

Clinical phenotype should be interpreted across current inflammation, cumulative remodeling, functional impairment, and treatment response. These dimensions may coexist and change over time; none can be inferred reliably from eos/hpf alone.

Endotyping seeks to explain this heterogeneity mechanistically. The same endoscopic phenotype can arise from different pathways, and the same pathway can generate different manifestations according to timing, tissue compartment, and accumulated remodeling.

The clinical heterogeneity described above is reflected in several practical clinical and disease-behavior patterns, summarized in **Table 1**.

**Table 1:** Practical clinical patterns and disease-control states in eosinophilic esophagitis.

Pattern/state	Clinical and endoscopic features	Histologic/structural profile	Behavior and biological context
Inflammatory	More common in children or early disease; feeding difficulty, vomiting, dysphagia; edema, furrows, exudates.	High epithelial eosinophilia, basal-zone hyperplasia, active injury.	Often reversible and treatment-responsive; IL-13/CCL26-dominant type 2 activity.
Fibrostenotic	Dysphagia or food impaction; rings, strictures, narrow caliber, reduced distensibility.	Variable eosinophilia; lamina propria fibrosis and altered compliance.	Cumulative and partly persistent; TGF-β, periostin, ECM, vascular–stromal and smooth-muscle remodeling.
Mixed	Inflammatory features coexist with rings, narrowing, or strictures.	Active eosinophilic inflammation plus structural remodeling.	Current inflammation and cumulative injury coexist; overlapping immune and structural programs.
Treatment-responsive	Symptomatic, endoscopic, and/or histologic improvement after PPI, topical corticosteroid, diet, or biologic therapy.	Reduced eosinophilia and epithelial injury; fixed lesions may remain.	A response pattern, not a separate endotype; indicates modifiable disease activity.
Relapsing/barrier-dominant	Recurrence after withdrawal or incomplete control; inflammatory endoscopy may be mild or variable.	Persistent or recurrent epithelial and barrier abnormalities.	Treatment-dependent course; barrier dysfunction, alarmins, antigen re-exposure, and residual molecular activity.
Remission under therapy	Few symptoms and improved EREFS; adaptive behavior or residual dysphagia may persist.	Eosinophils below the selected threshold; barrier, mast-cell, or remodeling abnormalities may remain.	Chronic disease control rather than cure; symptomatic, histologic, endoscopic, molecular, and structural remission may diverge.

## 7 Immunologic endotypes in EoE

Phenotypes describe how EoE presents and behaves; endotypes describe the biological programs proposed to generate that presentation. Only the EoEe1–EoEe3 transcriptomic classification has undergone formal multidimensional derivation, and even this framework is cross-sectional rather than a validated treatment-selection tool (7). The additional modules below should therefore be read according to their evidence level.

The best-established molecular endotyping framework is the EoEe1–EoEe3 classification, which integrates transcriptomic, clinical, endoscopic, and histologic features. EoEe1 is associated with a milder molecular and endoscopic pattern, EoEe2 with an inflammatory and often steroid-refractory pattern, and EoEe3 with adult-onset fibrostenotic disease and loss of epithelial differentiation programs. Importantly, these groups were not defined by proportional differences in eosinophil density, indicating that eosinophilia is a shared downstream feature rather than a complete descriptor of disease biology (7). Additional cytokine-expression profiling has shown that type 2 inflammation itself is heterogeneous, with variation in IL4, IL5, IL13, TSLP, CCL26, CCR3, CLC, and mast-cell-associated markers across active EoE cohorts (8).

Single-cell studies further support tissue-level heterogeneity. Inflammatory tissue T-cell populations enriched in EoE include GATA3-positive Th2-like cells capable of producing IL-5 and IL-13 (70). Clonally expanded GPR15-expressing pathogenic effector Th2 cells, including milk-reactive populations, suggest local antigen-associated immune organization within the esophagus (9). Mast-cell analyses also show heterogeneous and locally activated mast-cell populations, some of which may persist during histologic remission (44). These findings are consistent with clinical observations linking mast cell infiltration to persistent symptoms and endoscopic abnormalities despite resolution of eosinophilia (4).

An IL-13-high epithelial module is the most strongly supported proposed endotype. It links STAT6 signaling to CCL26, CAPN14, impaired differentiation, barrier dysfunction, basal-zone hyperplasia, and remodeling-associated gene expression (21, 26–35). Human tissue and interventional studies support the causal relevance of this axis.

An eosinophil–mast-cell effector module is supported by tissue, degranulation, treatment, and single-cell studies. It may explain active injury or persistent symptoms when granule-protein deposition, mast-cell mediators, neuroimmune signaling, or smooth-muscle effects are not reflected in counts of intact eosinophils.

An alarmin-driven epithelial-stress module links TSLP, IL-33, and IL-25 to Th2 cells, ILC2s, basophils, and downstream IL-13 activity (17–19, 71). Its proposed association with relapsing or trigger-sensitive disease is biologically plausible but remains hypothesis-generating because no validated clinical marker identifies this module in routine care.

A remodeling-dominant stromal–vascular module includes TGF-β-related fibrogenesis, periostin, extracellular-matrix deposition, endothelial–fibroblast crosstalk, smooth-muscle dysfunction, and reduced distensibility. Clinical, functional, and translational studies support this program, although prospective molecular classification remains unavailable (10, 12, 38, 47–59, 72, 73).

Treatment response is an operational disease dimension rather than a fixed biological endotype. PPI-, steroid-, diet-, and biologic-responsive patterns describe modifiable disease under a specific intervention; they should not be interpreted as separate diseases or as proof of a unique molecular mechanism (2, 68, 74, 75).

Most patients are likely to express overlapping programs whose relative dominance shifts with disease duration, therapy, relapse, and progression. Prospective longitudinal studies are required before these modules can be used for routine stratification.

## 8 Mapping immunologic endotypes to clinical phenotypes

Endotype-to-phenotype mapping is useful as an explanatory framework: similar eosinophil counts can accompany different epithelial, effector-cell, and structural programs. The relationships below are probabilistic and overlapping, not one-to-one assignments.

IL-13-high epithelial activity maps most closely to inflammatory and barrier-dominant disease, including edema, furrows, exudates, eosinophil recruitment, impaired differentiation, and basal-zone expansion. These features remain potentially modifiable by established anti-inflammatory therapy.

Eosinophil–mast-cell effector activity maps to active symptoms and residual tissue injury. Granule proteins, mast-cell mediators, neuroimmune signaling, or smooth-muscle effects may sustain dysphagia or endoscopic abnormalities after eosinophil counts decline.

Alarmin-driven epithelial stress may contribute to relapsing or trigger-sensitive disease, but this link remains mechanistic. Remodeling-dominant pathways map more directly to rings, strictures, low distensibility, food impaction, and the need for dilation.

Treatment-responsive patterns cut across all modules. Incomplete response may reflect persistent barrier dysfunction, mast-cell activity, or structural disease rather than failure to reduce eosinophilia alone. Current evidence does not support selecting PPI, corticosteroid, diet, or biologic therapy solely from a molecular endotype.

The EoEe1–EoEe3 classification provides formal evidence that clinical and endoscopic patterns do not map directly onto eosinophil density: EoEe1 is relatively mild, EoEe2 inflammatory and often steroid-refractory, and EoEe3 adult-onset and fibrostenotic (7).

Persistent periostin, DSG1 downregulation, basal-zone hyperplasia, or activated mast-cell programs during histologic remission illustrate why therapy may suppress one compartment while another remains active (10, 44, 76).

Overall, the framework should guide interpretation and hypothesis testing rather than rigid classification. Its clinical value will depend on prospective demonstration that a measurable endotype predicts response, relapse, or fibrostenotic progression better than standard multidimensional assessment.

**Figure 3:**
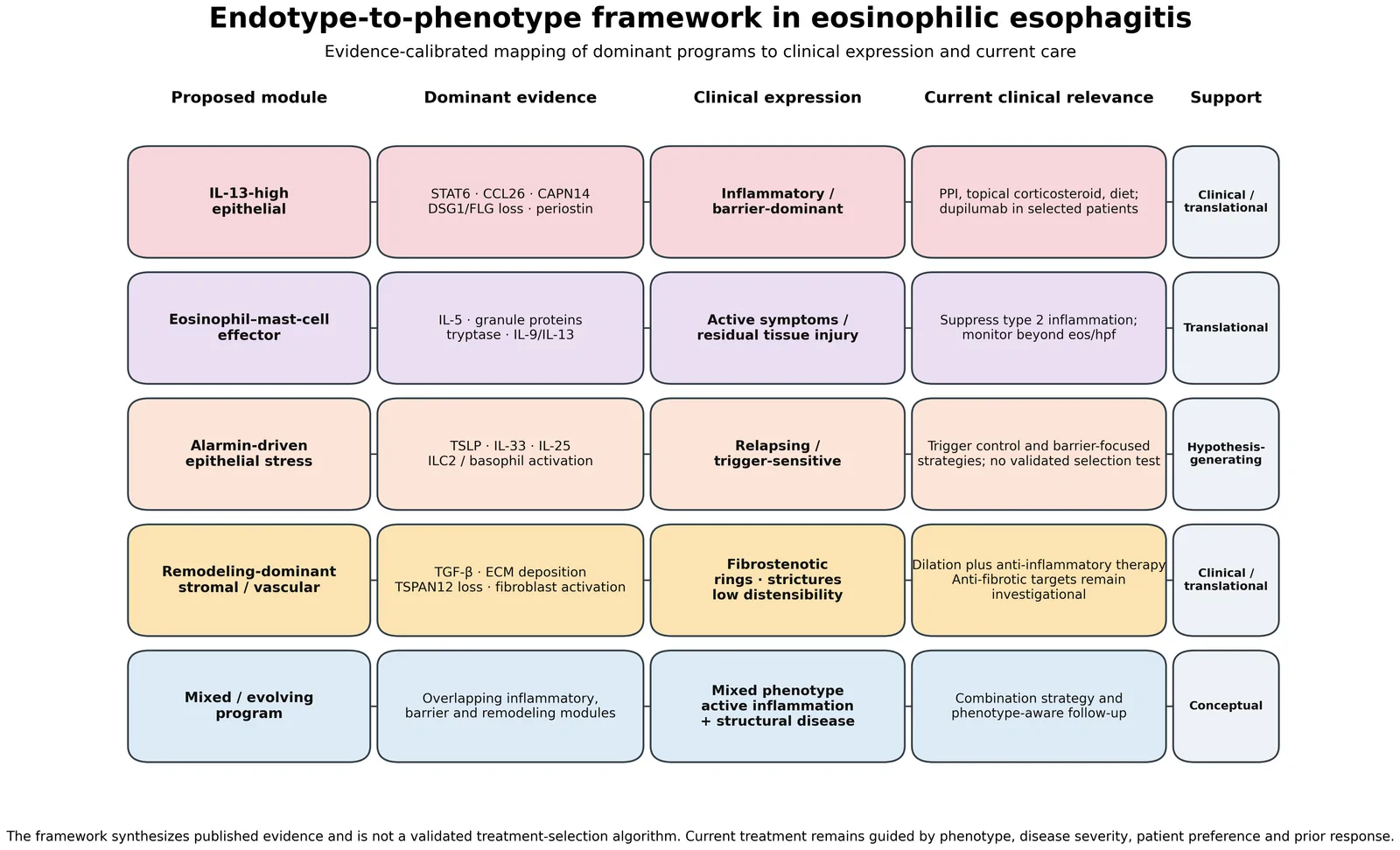
Evidence-calibrated endotype-to-phenotype framework in eosinophilic esophagitis. The image distinguishes clinically and translationally supported modules from hypothesis-generating constructs and explicitly positions PPIs among established treatments for inflammatory or barrier-dominant disease. The framework synthesizes published evidence and is not a validated treatment-selection algorithm.

The proposed relationship between dominant immunologic endotypes and clinical disease expression is summarized in **Figure 3**. A phenotype-oriented summary of the principal immunologic endotypes, dominant pathways, and potential clinical relevance is provided in **Table 2**.

**Table 2:** Proposed biological modules, clinical expression, and level of support in eosinophilic esophagitis.

Proposed module	Key evidence	Clinical expression	Clinical relevance/evidence level
IL-13-high epithelial	IL-13/STAT6; CCL26, CAPN14, DSG1↓, FLG↓, periostin, ANO1.	Inflammatory or barrier-dominant disease.	Established mechanistic axis with human tissue and treatment support; no validated biomarker-selection algorithm.
Eosinophil–mast-cell effector	IL-5, IL-9, granule proteins, tryptase, mast-cell activation.	Active symptoms or residual tissue injury.	Translational support; monitor beyond intact eos/hpf when domains are discordant.
Alarmin-driven epithelial stress	TSLP, IL-33, IL-25; ILC2 and basophil activation.	Relapsing or trigger-sensitive pattern.	Mechanistic and hypothesis-generating; no routine clinical marker.
Remodeling-dominant stromal/vascular	TGF-β, ECM, TSPAN12 loss, fibroblast and smooth-muscle activation.	Fibrostenotic disease with rings, strictures, and low distensibility.	Clinical and translational support; dilation plus anti-inflammatory therapy is established, anti-fibrotic matching is investigational.
Mixed/evolving	Overlapping inflammatory, barrier, effector, and structural programs.	Mixed phenotype and changing disease behavior.	Conceptual synthesis requiring longitudinal validation.

## 9 Why histology is not enough

Histology remains indispensable, but peak eosinophil count captures only one component of disease activity. Symptoms, EREFS, broader histologic injury, mast-cell and eosinophil effector activity, barrier dysfunction, and fibrostenotic remodeling overlap without resolving simultaneously.

The symptom–histology relationship is particularly weak. In adults, validated symptom instruments detect endoscopic or histologic remission with only modest accuracy, so absence of symptoms cannot reliably indicate biological remission (77). Pediatric studies also show dissociation between symptoms and histologic severity, with some patients symptomatic despite histologic improvement and others showing active eosinophilic inflammation with few symptoms (78). This discordance reflects adaptive eating behaviors, age-related reporting, neurosensory mechanisms, and mechanical effects of strictures. Dilation may improve dysphagia without reducing inflammation, further separating symptom response from histologic response (79).

Peak eosinophil count is limited because it captures acute epithelial eosinophilia but not the full histologic spectrum of EoE. The EoE Histology Scoring System was developed to address this by assessing basal zone hyperplasia, eosinophil abscesses, surface layering, dilated intercellular spaces, epithelial alteration, dyskeratosis, and lamina propria fibrosis (80, 81). These features provide information on epithelial injury and remodeling missed by eosinophil counts alone. Later studies confirmed that EoEHSS grade and stage correlate with endoscopic and histologic activity but not consistently with symptoms, showing that even expanded histology cannot fully represent all clinical domains (82).

Persistent epithelial abnormalities in histologically inactive disease further limit eosinophil-centered monitoring. Basal cell hyperplasia may persist when eosinophil counts fall below the diagnostic threshold and is associated with ongoing symptoms and endoscopic abnormalities (76). This suggests that epithelial repair may lag behind eosinophil clearance. Similarly, persistent desmoglein-1 downregulation and periostin accumulation in inactive or deep-remission EoE indicate that barrier dysfunction and remodeling signals may continue even when eosinophilic inflammation appears controlled (10).

Mast cells and eosinophil granule proteins add another layer of discordance. Mast cell activation may persist despite eosinophilia resolution and is associated with symptoms and endoscopic abnormalities (4, 44). Eosinophil granule protein deposition may also correlate with symptoms independently of intact eosinophil counts, suggesting that tissue injury can persist after standard histology no longer detects active cellular eosinophilia (39). Thus, eos/hpf may underestimate residual effector-cell activity when degranulation or mast-cell activation predominates.

Molecular studies reinforce this limitation by identifying distinct inflammatory and fibrostenotic programs at similar eosinophil densities (7, 8). Molecular profiling remains a research tool, but it shows that eosinophilia is a shared endpoint rather than a complete biological descriptor.

Remodeling is the domain least captured by peak eosinophil count. Rings, strictures, and reduced distensibility reflect cumulative structural injury rather than current mucosal eosinophilia. Distensibility studies and natural history data show that fibrostenotic burden relates strongly to disease duration and diagnostic delay, while histologic activity may fluctuate independently (54–57). Once fibrosis and altered mechanics are established, symptoms may be driven more by fixed narrowing and reduced compliance than by active epithelial eosinophilia.

EoE assessment should therefore integrate symptoms and adaptive behavior, peak eosinophil count and EoEHSS features, EREFS, and structural or functional assessment when indicated. Tissue-proximal biomarkers may eventually complement this framework, but none currently replaces biopsy in routine care.

### 9.1 Remission is domain-specific and usually treatment-dependent

In this review, remission denotes control of a chronic disease, usually during ongoing therapy, rather than cure. Symptomatic remission refers to the absence of symptoms or a clinically meaningful improvement in symptoms after accounting for adaptive eating behavior. Histologic remission is commonly defined clinically as <15 eos/hpf, although stricter thresholds are used in trials. Endoscopic remission refers to marked improvement or resolution of EREFS activity; fixed rings or strictures may persist. Molecular remission denotes normalization of disease-associated tissue signatures and remains investigational. Structural or functional remission requires improvement in caliber, fibrosis, compliance, or distensibility and may lag behind inflammatory control.

These domains should be reported separately because symptomatic, histologic, endoscopic, molecular, and structural responses are not interchangeable. Maintenance therapy and structured follow-up are generally required because treatment withdrawal can be followed by recurrence and because ongoing inflammation may permit remodeling (1, 62, 83, 84).

## 10 Discussion

### 10.1 Therapeutic implications: phenotype-aware care and future endotype testing

Current EoE treatment is phenotype-aware rather than molecularly endotype-guided. Selection is based on symptoms, EREFS and histology, inflammatory versus fibrostenotic disease, patient preference, dietary feasibility, treatment availability, comorbid atopy, and prior response. PPIs, swallowed topical corticosteroids, dietary elimination, dupilumab, and dilation have established clinical roles, whereas alarmin-, mast-cell-, barrier-, biomarker-, and anti-fibrotic matching remain investigational (1, 85–90).

**Table 3** summarizes the principal mechanisms and clinical positioning of established therapies. These interventions act across overlapping pathways and should not be assigned to a single endotype.

**Table 3:** Established therapies and their principal mechanisms in eosinophilic esophagitis.

Therapy	Principal mechanism	Established clinical role	Endotype/phenotype interpretation
Proton pump inhibitors	Acid suppression plus epithelial and anti-inflammatory effects, including reduced eotaxin-3 signaling and improved barrier function.	First-line induction and maintenance option in appropriate patients.	Response defines a treatment-response pattern, not a separate disease or validated endotype.
Swallowed topical corticosteroids	Broad local suppression of type 2 cytokines, eosinophilic inflammation, and epithelial injury.	Established induction and maintenance therapy for inflammatory or mixed disease.	Mechanistically broad; steroid-refractory disease does not identify one specific endotype.
Empiric elimination diet	Reduces food-antigen stimulation upstream of local type 2 inflammation.	Established non-pharmacologic induction and maintenance strategy.	Supports trigger-responsive disease; sensitization testing alone does not reliably identify causal foods.
Dupilumab	IL-4Rα blockade inhibits IL-4 and IL-13 signaling and downstream epithelial programming.	Biologic option for selected patients with active EoE.	Strong type 2 rationale, but no validated molecular biomarker is required or established for selection.
Esophageal dilation	Mechanical restoration of luminal caliber without direct anti-inflammatory activity.	Adjunct for strictures, narrow-caliber esophagus, or persistent mechanical dysphagia.	Targets the fibrostenotic phenotype and should be combined with anti-inflammatory disease control.
Emerging strategies	Alarmin, mast-cell, barrier-restoring, and anti-fibrotic targets.	Clinical trials and translational research.	Candidate endotype matching remains hypothesis-generating.

Mechanistic interpretation remains useful. PPIs can influence acid exposure, epithelial barrier function, and type 2 inflammatory signaling; topical corticosteroids broadly suppress mucosal inflammation; diet removes upstream antigenic stimulation; dupilumab blocks IL-4/IL-13 signaling; and dilation treats mechanical narrowing without controlling inflammation. These mechanisms explain response patterns but do not yet justify molecular matching in routine care.

Routine molecular endotyping of every patient is not currently justified. No prospective algorithm has shown superior outcomes, standardized assays and clinically interpretable thresholds are lacking, and cost-effectiveness has not been established. Many patients can be managed with established therapies using multidimensional clinical assessment (1, 62, 84).

The potential incremental value of endotypic characterization is greatest in research settings and in selected difficult cases: repeated treatment failure, discordant symptoms and histology, persistent molecular or epithelial injury, progressive fibrostenosis, or consideration of future targeted therapies. Even in these settings, endotyping should complement rather than replace EREFS, histology, functional assessment, and treatment history.

### 10.2 Future directions: validating biomarkers and stratified care

The immediate research priority is not routine endotyping, but prospective validation of whether measurable biological programs improve prediction beyond symptoms, EREFS, histology, and structural assessment. Longitudinal studies should distinguish markers of current inflammation from predictors of relapse, treatment response, and fibrostenotic progression.

Tissue transcriptomics and the EoE Diagnostic Panel demonstrate that molecular activity can be measured beyond peak eosinophil counts (91, 92). Their clinical role will depend on simplified assays, external validation, reproducible thresholds, and evidence that molecular information changes management or outcomes.

Single-cell studies are refining epithelial, immune, stromal, and myeloid states during active disease and remission (44, 70, 93). These datasets may identify persistent cell programs after eosinophil reduction, but they remain discovery platforms rather than routine tests.

Multi-omics integration may generate composite signatures, but current studies remain limited by small cohorts, cross-sectional sampling, technical variability, and incomplete external validation (94–96).

Reliable non-invasive or minimally invasive monitoring remains a major unmet need because repeated endoscopy with biopsy is burdensome and costly (97). Circulating eosinophils, cytokines, and granule proteins have insufficient stand-alone accuracy, whereas tissue-proximal approaches—especially the esophageal string test, brushing, and Cytosponge-based sampling—generally align more closely with mucosal inflammation (62, 98). These tools may reduce endoscopy burden in selected follow-up scenarios, but they require prospective validation within defined algorithms and cannot replace endoscopy when diagnosis is uncertain, symptoms and biomarkers are discordant, or structural complications are suspected.

Future trials should predefine clinical phenotype, EREFS, EoEHSS, distensibility, and candidate molecular modules before treatment and test whether these variables predict benefit from PPI, topical corticosteroid, diet, or biologic therapy. Such studies should include health-economic analyses and clinically actionable decision thresholds.

Patient-derived organoid and ex vivo systems can test epithelial differentiation, cytokine responses, barrier repair, and candidate therapies (99). Their value is presently mechanistic and preclinical.

Implementation requires assay standardization, multicenter validation, transparent prediction models, bioinformatics infrastructure, and demonstration of clinical utility and cost-effectiveness. Classification accuracy alone is insufficient.

The realistic near-term goal is a multidimensional treat-to-target framework that distinguishes inflammatory control, epithelial repair, endoscopic response, and structural recovery. Molecular endotyping may eventually refine this framework, particularly in refractory or progressive disease, but it should not be portrayed as an established standard.

### 10.3 Limitations

This review has several limitations. It is a narrative, mechanism-oriented synthesis without formal PRISMA selection, risk-of-bias assessment, or quantitative evidence grading. The proposed endotype-to-phenotype framework is conceptual and has not been prospectively validated for treatment selection. Many molecular studies are cross-sectional, use single-time-point biopsies, or include small specialist cohorts. Standard mucosal biopsies may underestimate patchy inflammation, subepithelial remodeling, degranulation, mast-cell activity, and fibrostenotic burden. Molecular and minimally invasive tools are not standardized or routinely available, and cost-effectiveness data are sparse. The framework is derived exclusively from published evidence and should be tested in longitudinal, treatment-stratified cohorts.

## 11 Conclusion

EoE is a heterogeneous epithelial–immune and structural disease. Eosinophils remain diagnostically central and biologically active, but they operate within broader alarmin, Th2/ILC2, mast-cell, epithelial, stromal, and remodeling networks. This explains why eos/hpf, symptoms, EREFS, molecular activity, and structural impairment often diverge.

IL-13 provides a central mechanistic bridge through CCL26 induction, impaired differentiation, CAPN14 and DSG1-related barrier dysfunction, basal-zone hyperplasia, periostin, and remodeling. Eosinophil granule proteins, mast cells, TGF-β-dependent myofibroblast activity, endothelial–fibroblast interactions, and extracellular matrix organization add partially independent injury pathways.

Disease control should therefore be reported across separate domains: symptomatic, histologic, endoscopic, molecular, and structural or functional remission. In routine care, symptoms, EREFS, histology, and structural assessment remain primary; minimally invasive biomarkers are promising adjuncts but do not yet replace biopsy.

The proposed endotype-to-phenotype model is an evidence-organizing framework, not a validated treatment algorithm. Current therapy should remain phenotype-aware and response-based, while molecular characterization is most appropriately studied in refractory, discordant, or progressive disease.

Future multicenter studies should integrate symptoms, adaptive behavior, EREFS, EoEHSS, distensibility, transcriptomics, tissue-proximal biomarkers, treatment response, and health-economic outcomes. The key test is whether an endotype is stable, measurable, modifiable, and clinically predictive beyond standard multidimensional assessment.
